# Sex-Specific Association between Childhood BMI Trajectories and Asthma Phenotypes

**DOI:** 10.1155/2018/9057435

**Published:** 2018-12-02

**Authors:** Danny Wadden, Leigh-Anne Allwood Newhook, Laurie Twells, Jamie Farrell, Zhiwei Gao

**Affiliations:** Faculty of Medicine, Memorial University of Newfoundland, St. John's, NL, Canada

## Abstract

**Background:**

Asthma and obesity are two common health problems in the pediatric population. Obesity is associated with several comorbidities which are of great consequence. Excess adipose tissue has been linked to asthma in a number of studies. However, little is known about childhood body mass index (BMI) trajectories and the development of asthma phenotypes.

**Objective:**

The current study aims to investigate the significance of BMI trajectories over childhood and the risk of asthma phenotypes.

**Methods:**

The current study is a prospective cohort of children aged 0-2 years who were followed every two years for eight years through cycles one to five in the National Longitudinal Survey of Children and Youths (NLSCY). Statistical analysis: a latent class growth modelling (LCGM) method was used to identify BMI trajectory patterns from cycles one to five. Multiple imputation (number of imputations=5) was carried out to impute children with missing values on height or weight information. Sampling weights and 1,000 bootstrap weights were used in SAS* PROC SURVEYLOGISTIC *to examine the association between BMI trajectory and asthma phenotypes (persistent or transient asthma) in a multivariate analysis.

**Results:**

The study consisted of 571,790 males and 549,230 females. Among them, 46% of children showed an increasing trajectory in terms of change in BMI percentile during childhood, followed by the stable-trajectory group (41%) and decreasing-trajectory group (13%). After controlling for confounding factors, females in the increasing BMI trajectory group were four times more likely to be associated with persistent asthma (OR = 4.09; 95% CI:1.04-16.15; p = 0.0442) than females in the stable BMI trajectory group. No such relationship was found in males. The BMI trajectory was not significantly associated with risk of transient asthma for either sex.

**Conclusion:**

We report a female-specific association between increasing adiposity, measured by BMI, and persistent asthma.

## 1. Introduction

Asthma is an airway disease characterized by inflammation, variable airflow obstruction, and symptoms including cough, wheeze, dyspnea, and chest tightness [1]. The World Health Organization (WHO) estimates that about 235 million people worldwide have a diagnosis of asthma [2]. The Canadian Thoracic Society (CTS) has published guidelines regarding both the diagnosis and management of asthma for both children and adults [3, 4]. The preferred method for the diagnosis in children older than six years and adults is showing reversible airway obstruction during spirometry; however alternative methods include variability of peak expiratory flow, methacholine challenge, or exercise challenge [3]. For children aged one to five years, the CTS published an algorithm which incorporates a clinical diagnosis based on symptoms and signs and a therapeutic trial of inhalers [4]. Treatment of asthma is important as it improves lung function, prevents hospitalizations, and reduces mortality [5–8].

The WHO estimates that globally obesity, or the excessive accumulation of adipose tissue, has almost tripled since the mid-1970s [9]. Children have also been affected by the obesity epidemic. In 2016, the WHO reported on overweight/obesity in children younger than five years of age and for children and adolescents between 5 and 19 years of age; 41 million and 340 million were either overweight or obese for the each cohort, respectively. [9]. Obesity is estimated by body mass index (BMI), which is a person's weight in kilograms, divided by height in metres squared [10]. In children, obesity is defined as a value above the 95^th^ percentile for BMI matched for age and sex, while the definition of overweight is defined as a value above the 85^th^ percentile matched for the same variables [11]. Obesity has been shown to be associated with respiratory diseases including obstructive sleep apnea, obesity hypoventilation syndrome, and asthma [12–14].

The link between obesity and asthma has been studied; having more adiposity, represented by an increasing BMI, has been shown in many studies to be associated with a greater incidence of asthma [15–18]. Additionally, it has been found that asthma severity is positively associated with obesity [19, 20]. Individuals who have asthma and are living with obesity are more challenging with regard to controlling the disease process [21–23]. The relationship between obesity and asthma/related symptomatology has also been demonstrated in the pediatric population [24, 25].

Because childhood obesity has become more prevalent [26], it is important to understand the natural course of this process. [27]. A study conducted by Chen et al. identified six specific trajectory groups based on BMI z-score and five trajectories when separating overweight/obese children, in a cohort of 1651 elementary school children followed from kindergarten to grade five [28]. The researchers observed the following trajectories when separating the obese children into three groups: persistent obesity, becoming obese, and chronic obesity [28]. Moreover, few studies have investigated the relationship between BMI trajectories and asthma. Rzehak et al. published results from eight birth cohorts in Europe and found a BMI which rapidly increased between birth and two years was associated with a risk of asthma up to age six [29]. Other studies have defined obesity patterns which have been associated with asthma [30–32]; however the associations between childhood BMI trajectory and risk of asthma phenotypes, specifically sex-specific, are still lacking. As a result, using a Canadian national childhood cohort, we sought to ask the following research objectives: (1) to describe the prevalence of persistent asthma and transient asthma; (2) to identify BMI trajectory patterns over childhood; (3) to examine the sex-specific association between BMI trajectory patterns and the risk of different asthma phenotypes, specifically persistent asthma.

## 2. Methods

### 2.1. Ethics Approval

The study was approved by the provincial Health Research Ethics Authority (HREA), at Memorial University in St. John's, NL, Canada.

### 2.2. Study Population

The National Longitudinal Survey of Children and Youths (NLSCY) cycles one to five were used for our study. This longitudinal survey follows children from birth to early adulthood to monitor their health and wellbeing. The first cycle was launched by Statistics Canada in 1994 and consisted of 16,903 children aged 0 to 12 years from 10 provinces. Of the total sample, only children aged 0-2 years in the first cycle were included in our current longitudinal child cohort [33]. Information was gathered through interviews every two years (cycle 2: aged 2-4 years, cycle 3: aged 4-6 years, cycle 4: aged 6-8 years, and cycle 5: aged 8-10 years). Cycle 1 consisted of 6,660 children aged 0-2 years (mean: 1.02 years and standard deviation: 0.81). Out of these 6,660 children, 5,040 (76%) children were followed up in cycle 2 (aged 2-4 years); 4,900 (74%) children were followed up in cycle 3 (aged 4-6 years); 4,400 (66%) children were followed up in cycle 4 (aged 6-8 years); 4,255 (64%) children were followed up in cycle 5 (aged 8-10 years).

### 2.3. Outcome Variables: Asthma Phenotypes

Asthma phenotypes (persistent and transient asthma) were determined by the responses to the following two questions from the person most knowledgeable (PMK) of the child: (1) Ever-asthma- “Does this child have asthma that has been diagnosed by a Health Professional”; (2) Current-asthma- “Has he/she had an attack of asthma in the last 12 months?” Persistent asthma was defined as a child having a positive response on the Ever-asthma or Current-asthma question in the earliest cycle and positive responses on the Current-asthma question in ALL the subsequent cycles. Transient asthma was defined as a child having a positive response on the Ever-asthma or Current-asthma question in the earliest cycle and no report of Current-asthma in ALL the subsequent cycles.

### 2.4. Main Exposure Variable: BMI Trajectory

BMI [(kg)/height(m^2^)] was calculated for each child. The different BMI growth patterns in childhood (from cycle one to cycle five) were based on the child's sex- and age-specific BMI 20-quantiles (ventiles) ranging from 0 to 19: 0 (less than 5 percentile) and 1 (between 5 and 10 percentile) to 18 (between 90 and 95 percentile) and 19 (more than 95 percentile). For children with missing information on height and weight, multiple imputations (MI) were used to impute missing values. The multiple imputation method has been described in detail by Allison [34]. This method has many advantages over conventional methods including list-wise deletion, pair-wise deletion, and imputations using marginal means or regression models since these common methods generally introduce substantial bias by ignoring the between-imputation variability, and yield smaller standard errors, thus increasing the likelihood of statistical significance. In the current study, multiple imputations (number of imputation = five) for missing values were carried out by SAS® Proc MI [35].

### 2.5. Other Risk Factors

The following child, parental, and household level factors were considered in this study:  *Predictors of asthma during pregnancy and early life* include age of child, sex of child, premature delivery (<=258 days);  *Parental predictors of asthma* include PMK and spouse current smoking status, highest level of education for PMK and spouse, PMK total annual income, biological mother history of asthma, age of biological mother/father at birth of child, PMK/spouse number of cigarettes per day, and Household Socio-Economic Status (HSES). The HSES was derived by Statistics Canada from the following five sources for each household: the level of education of the PMK, the level of education of the spouse/partner, the prestige of the PMK's occupation, the prestige of the occupation of the spouse/partner, and household income [33]; *Household predictors* include dwelling ownership, and dwelling in need of repair.

### 2.6. Statistical Analysis

To identify distinct change patterns of BMI during childhood, a latent class growth modelling (LCGM) method was carried out by SAS®* Proc Traj* in this study [36, 37]. LCGM method able to capture the heterogeneity of subgroups among a population by simultaneously estimating several trajectories as opposed to fitting an overall population mean [36, 37]. In order to find the optimal number of trajectories, Bayesian Information Criterion (BIC) was used to compare the fitness of models between trajectories with different number of groups or between different shapes of a trajectory [36, 37]. We also defined a minimum of 10% for each of the trajectory groups. Mean (standard deviation, STD) and count (frequency) were calculated for continuous and categorical variables, respectively. Sampling weights were included in all statistical analyses.* PROC SURVEYLOGISTIC* was used to identify the significant risk factors for each of the asthma phenotypes (persistent or transient asthma) in the univariate analysis and to examine the association between BMI trajectory and asthma phenotypes in the multivariate analysis. Only clinically important factors and variables with a p-value lower than 0.20 in the univariate analysis were included in the multivariate analysis. Variables such as mother's age at birth, mother's history of asthma, household smoking, and socioeconomic-related variables (i.e., education level and income) which have all been studied in relation to childhood asthma risk [38–42]. To account for complex survey design, variances were estimated using 1,000 bootstrap weights, which were provide by Statistics Canada. The level of significance *α* = 0.05 was used for the multivariate logistic regression. Due to the multiple imputation of BMI (number of imputation = five) in our study, SAS®* Proc MIanalyze* was used to combine the five sets of regression coefficients. Data analysis was conducted using SAS® version 9.4.

## 3. Results

**Figure 1:**
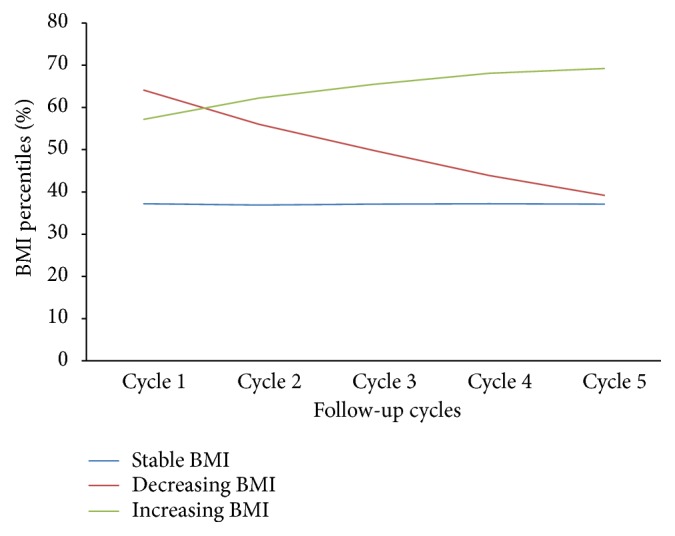
BMI trajectory groups from cycle 1 to 5.

Population characteristics are described in Table 1. The cohort analyzed consisted of 1,121,020 participants: females (49%) and males (51%). The average age of children at baseline was 1.02 years (STD = 0.81). The three defined BMI trajectory groups are shown in Table 1 and Figure 1; stable BMI (41%), decreasing BMI (13%), and increasing BMI (46%). During the 8 years of follow-up, the prevalence of persistent and transient asthma were 3% and 13%, respectively. At the beginning of follow-up (cycle 1), 24% of children lived in a house in which the PMK smoked, 27% in which the PMK's spouse smoked and 23% in which both PMK or PMK spouse smoked. In houses in which PMK or spouse smoked, 45% of children lived in houses in which both PMK and spouse smoked. With regard to home ownership, 66% of children lived in a home that was owned. Major home repairs needed were reported in 7% of respondents, while minor repairs needed and no repairs needed were reported as 16% and 78%, respectively. The highest level of education of respondent was reported as the following- secondary school or less: 21%; beyond high school: 26%; college/university or higher: 54%. Ten percent of children were reported as being born premature. Six percent of children had a mother who had a history of asthma. The PMK income was reported as the following: less than $15,000, 54%; $15-30,000: 25%; greater than $30,000: 21%. Seven percent of children were reported to have an allergy. With regard to ethnicity, 75% of the population were white.

The univariate analyses for both the persistent and transient asthma groups are described in Table 2. Being female was significantly less likely to be associated with having persistent asthma compared to being male (OR = 0.51, 95% CI: 0.296-0.88, p = 0.0157). Also, compared with a PMK annual income >$30,000, children of PMKs with less than $15,000 annual income were more than two times more likely to be associated with having persistent asthma (OR = 2.49, 95% CI: 1.22-5.06, p = 0.012). Children of PMKs who were current daily smokers were at significantly higher risk of transient asthma than children of PMKs who were not current smokers (OR = 1.61, 95% CI: 1.18-2.19, p-value = 0.0025). Compared with children of PMK and spouses with education level of university/college, being a child of PMK and spouses with an education beyond high school group or with the education of the secondary school and less was significantly associated with higher risks of transient asthma (OR_secondary  school_ = 1.4, 95% CI: 1.01-1.95, p-value = 0.0439; OR_less  secondary_ = 1.54, 95% CI: 1.04-2.3, p-value = 0.0319). PMK number of cigarettes per day was also significantly associated with a higher risk of transient asthma (OR = 1.02, 95% CI:1.00-1.04, p = 0.0186). Being born from a mother with asthma was significantly more likely to be associated with transient asthma than children of mothers without a diagnosis of asthma (OR = 2.33, 95% CI: 1.32-4.13, p = 0.0038). Being a child with an allergy was significantly more likely to be associated with both persistent (OR = 3.10, 95% CI: 1.44-6.66, p = 0.0038) and transient asthma (OR = 1.85, 95% CI: 1.21-2.81, p = 0.0043, compared to children without allergies. Age of child at baseline was associated with a lower risk of persistent asthma (OR = 0.68, 95% CI: 0.50-0.95, p = 0.0212) and a higher risk of transient asthma (OR = 1.29, 95% CI: 1.07-1.55, p = 0.0065). No significant association was observed for ethnicity.

Table 3 shows the results of sex-specific multivariate analyses. Females in the increasing BMI trajectory group were about four times more likely to be associated with persistent asthma than females in the stable BMI trajectory group (OR = 4.09, 95% CI: 1.04-16.15, p = 0.0442) after controlling for potential confounders. No such association was found for males with persistent asthma. BMI trajectory group was not significantly associated with the transient asthma for either sex.

## 4. Discussion

The most importance finding from the present study was a sex-specific association between childhood BMI trajectory and persistent asthma. After controlling for confounders, girls within the increasing BMI trajectory group had a more than 4 times greater risk of persistent asthma than girls in the stable BMI trajectory group. This relationship was not found for boys. BMI trajectory group was not significantly associated with risk of transient asthma for either sex. These findings show a correlation between obesity and an asthma phenotype, which is sex-specific.

Previous studies have found sex-specific associations between obesity and asthma. A prospective cohort study using data from the Nurses' Health Study II showed the relative risk of asthma was higher with increased BMI [43]. Chen at al. using data from 9,149 Canadians (4,266 males, 4,883 females) found that in women baseline BMI predicted asthma [44]. No such significance results were found for male subjects. A Canadian paper by Wang et al. also using the NPHS studied adults aged 40-55 years and developed four trajectories based on BMI [45]. The manuscript focused on stable trajectories: normal, overweight, and obese class I and class II. The authors found that the obese stable II (higher BMI) group had a higher reporting of asthma (OR = 2.6) [45]. However, Stanley et al., used the First Health and Nutrition Examination Survey and published data from 14,407 participants aged 25-74 with a diagnosis of asthma [31]. They stated that a BMI which increased was correlated with asthma prevalence only at baseline and follow-up and not during the observation interval. Nevertheless, other analyses showed the relative risks (with 95% confidence intervals) of asthma in elevated, markedly elevated, and severely elevated BMI groups compared to the normal were 1.0 (0.9-1.2), 1.0 (0.8-1.3), and 1.1 (0.8-1.5), respectively [31].

With regard to the pediatric population, very few studies exist regarding BMI trajectories and asthma. A study from 2014 which used a birth cohort from 1456 individuals born from January 1989-Febuary 1990 observed four different BMI trajectories from age 1-18 years: normal, early persistent obesity, delayed overweight and early transient overweight [32]. The early persistent obesity group had a 2.15-fold risk of asthma compared to the normal trajectory group at the age of 18 years. Chen et al. published data from the Taiwan Children Health Study using 4422 subjects aged 6-11 years [30]. They questioned if various defined growth trajectories were associated with the development of asthma and rhinitis at 12, 15, and 18 years of age. The growth trajectories defined were normal growth, rapid growth, persistently overweight and declining obesity [30]. Interestingly, at age 12, the persistent overweight group had an increased asthma incidence. The study which most mirrored the current study was a Swedish Study that investigated 2818 children from data collected between 1994 and 2013 [46]. BMIs were calculated, and participants with asthma were described as transient, persistent, or late-onset. The female participants with persistent asthma had a greater odds of being overweight (BMI > 85^th^ percentile) compared to their nonasthmatic counterparts. The authors noted this difference was greater with age, and no such associations were present in the male subjects [46]. The sex-specific findings from both this study and the present question if sex plays a role in the association between adiposity and asthma.

Explanations for why a sex-specific difference exists regarding obesity and asthma have been explored previously. Physical activity may contribute toward this relationship as the boys have been documented to be more physically active as compared to girls [47, 48]. Lochte et al. reported that low physical activity levels may be associated with a risk of new onset asthma in children and adolescent [49]. We feel asthmatics may not take part in as much physical activity, due to respiratory issues, and therefore may have a less favorable body composition. Additionally, it is known that females tend to go through puberty earlier, and have more adiposity during this period [50]. It could be theorized that a hormonal difference between males and females may be at play. Interestingly, body fat has been associated with levels of estrogen [51]. In girls, estrogen is thought to influence body composition during puberty [52]. Another potential hormone effect is luteinizing hormone (LH); as described by McCartney et al. in both prepubertal and early puberty girls adiposity was related to reduced secretion of LH, while in later pubertal girls, increased LH frequency and decreased amplitude were associated with adiposity [53]. Evidently, further studies are necessary to understand the sex-specific mechanism relating obesity and asthma in girls.

Obesity linked asthma phenotypes have been described; Moore et al. determined five novel asthma phenotypes in a study of 726 subjects [54]. One of these was a cluster of 59 subjects characterized by late-onset nonatopic asthma in older obese females (mean age of 50). Over half of these subjects were receiving ≥3 asthma drugs, while 17% stated they used oral corticosteroids, representing a cohort with poorly controlled asthma [54]. Similarly, Haldar et al. observed an obese, noneosinophilic cluster of asthmatics in both a cohort of mainly mild to moderate asthma (n = 184) and a secondary care cohort consisting of refractory asthma [55]. Though our cohort was all children, we question if obese females, regardless of age, are at a higher risk of asthma.

The mechanistic relationship between obesity and asthma has been theorized. Obesity is thought to induce a proinflammatory state and adipose tissue releases signals including leptin, TNF-alpha and IL-6 [56–59]. The exact mechanism of phenotypes of asthma in with obesity have been questioned to involve T_H_2 cytokines as CD4+ cells are important in asthma inflammation [60]. However, Dixon et al. looked at 44 participants (23 asthmatics and 21 nonasthmatics) in relationship to bariatric surgery and various outcome measures [61]. They noted improved control, improved methacholine airway responsiveness and quality of life in asthmatics after surgery. However at 12 months, when analyzing the bronchoalveolar lavage fluid, the lymphocytic proportion was increased. Also increased were CD4+ derived cytokines (activated from blood), including IFN-gamma, TNF-alpha, IL-5, 6, 13 and 17 [61]. As these cytokines are related to inflammation, the authors felt that the improvement of asthma from decreasing adiposity was due to another novel pathway. A unique study of 40 patients with severe asthma looked at bronchial brushings and found subjects who were obese had a different airway microbiome composition then nonobese subjects [62]. Additionally, fewer eosinophils on bronchial biopsy were found in the obese group compared to nonobese. Nonetheless, while the exact mechanism of asthma with obesity phenotype may be multifactorial, the GINA considers the phenotype to include asthmatic patients with increased adiposity and minimal eosinophilic inflammation of the airways [1].

The present study has some limitations. Our study population was from a national survey, which represented only children currently residing in Canada. Additional studies on other pediatric populations in other parts of the world would help to further confirm the sex-specific link between BMI trajectories and asthma phenotype. Although the data used were self-reported, a meta-analysis of 23 studies has demonstrated self-reported BMI to be a feasible alternative to directly measured anthropometrics when such data is unavailable; sensitivity of 0.76 and specificity of 0.96 [63]. It is known that using BMI to estimate obesity can misclassify some individuals [64]. However, BMI is critical to such large population-based studies and more technical measures would be costly and time consuming. Strengths of the current study include its very large sample size, obtained through robust recruitment, representative of the Canadian childhood population.

Overall, using the NLSCY, we observed a sex-specific relationship between increasing BMI trajectory and persistent asthma in females only, after controlling for potential confounding factors. Other studies have shown a sex-specific relationship between obesity and asthma in children [46]. Whether sex-specific hormones play a role in persistent asthma with obesity requires further exploration. Future studies are needed to replicate this study finding and are necessary to further understand the mechanisms that may explain the relationship between asthma and obesity and the potential sex-specific association.

## Figures and Tables

**Table 1:** Descriptive statistics of study population (n = 1,121,020).

Variable		%
BMI Trajectory group	Stable BMI	41
	Decreasing BMI	13
	Increasing BMI	46

Persistent Asthma Status	Persistent Asthmatic	3
	Non- Persistent Asthmatic	97

Transient Asthma Status	Transient Asthmatic	13
	Non-Transient Asthmatic	87

Sex of Child	Female	49
	Male	51

Child age at baseline (Mean ± STD)	1.02 ± 0.81	
		

Child allergy status	Child With Allergy	7
	Child Without Allergy	93

Ethnicity	White	75
	Other	25

PMK^*∗*^ Smoking	Current Smoker	24
	Non-Smoker	76

PMK's Spousal Smoking	Current Smoker	27
	Non-Smoker	73

PMK or Spousal Smoking	Current Smoker	23
	Non-Smoker	77

House Ownership of Respondent	Home Owner	66
	Non Home Owner	34

Home Repair Needed	Major Repairs	7
	Minor Repairs	16
	No repairs	78

Highest Level of Education	Secondary School or Less	21
	Beyond High school	26
	College/University or Higher	53

Premature Birth of Child	Premature	10
	Non-premature	90

PMK Income	<$15,000	54
	$15,000- $30,000	25
	>$30,000	21

Mother's (biol) History of Asthma	Maternal Asthma	6
	No Maternal Asthma	94

^*∗*^PMK = person most knowledgeable.

**Table 2:** Univariate analysis within persistent and transient asthma groups.

		Persistent Asthma					Transient Asthma				
				95% CI					95% CI		
Variable		Prevalence (%)	OR	Low	High	P-value	Prevalence (%)	OR	Low	High	P-value
BMI Trajectory Group	1 (Stable BMI)	3	1				12	1			
	2 (Decreasing BMI)	2	0.28	0.172	1.944	0.37	13	1.08	0.53	2.23	0.82
	3 (Increasing BMI)	3	1.06	0.552	2.022	0.87	14	1.18	0.86	1.61	0.32

Sex of Child	Female	2	0.51	0.296	0.88	***0.0157***	11	0.73	0.54	1	0.05
	Male	4	1				15	1			

PMK Daily Smoking	Current Smoker	2	0.835	0.437	1.596	0.58	17	1.61	1.18	2.19	***0.0025***
	Non-Smoker	3	1				12	1			

PMK's Spousal Daily Smoking	Current Smoker	3	1.118	0.61	2.048	0.72	13	1.07	0.77	1.5	0.68
	Non-Smoker	3	1				12	1			

PMK + Spousal Daily Smoking	Current Smoker	3	1.256	0.685	2.3	0.46	13	1.01	0.72	1.39	0.99
	Non-Smoker	3	1				13	1			

House Ownership of Respondent	Home Owner	3	1.2	0.617	2.332	0.59	13	0.95	0.69	1.31	0.76
	Non Home Owner	2	1				13	1			

Home Repair Needed	Minor Repairs	4	1.487	0.396	3.176	0.31	11	0.83	0.57	1.2	0.33
	Major Repairs	3	1.261	0.593	2.68	0.55	14	1.1	0.7	1.74	0.67
	No repairs	2	1				13	1			

Highest Level of Education	Secondary School or Less	2	0.636	0.235	1.721	0.37	16	1.54	1.04	2.3	***0.0319***
	Beyond Highschool	2	0.713	0.381	1.336	0.29	15	1.4	1.01	1.95	***0.0439***
	College/University or Higher	3	1				11	1			

Premature Birth of Child	Premature	3	1.023	0.433	2.413	0.96	16	1.327	0.85	2.08	0.22
	Non-premature	3	1				13	1			

PMK Income	<$15,000	3	2.49	1.222	5.061	***0.0120***	13	0.787	0.531	1.168	0.23
	$15,000- $30,000	3	2.27	0.98	5.234	0.06	12	0.7	0.463	1.057	0.09
	>$30,000	1	1				16	1			

Mother's (biol) History of Asthma	Maternal Asthma	5	2.092	0.878	4.988	0.10	24	2.33	1.316	4.127	***0.0038***
	No Maternal Asthma	3	1				12	1			

Child Allergy Status	Child With Allergy	7	3.10	1.441	6.658	***0.0038***	21	1.846	1.212	2.81	***0.0043***
	Child Without Allergy	2	1				12				

Ethnicity	White	3	1.11	0.546	2.274	0.77	14	1.283	0.835	1.971	0.25
	Other	3	1				11	1			

Child Age at baseline			0.68	0.495	0.945	***0.0212***		1.289	1.074	1.548	***0.0065***

Household Socio-economic Status			1.115	0.782	1.591	0.55		0.859	0.71	1.039	0.12

Biol. Mother Age at Birth			1.042	0.987	1.1	0.14		0.984	0.952	1.017	0.35

Biol. Father Age at Birth			1.034	0.99	1.08	0.14		0.99	0.96	1.01	0.36

PMK daily cigarettes			0.996	0.953	1.042	0.87		1.02	1	1.04	***0.0186***
Spouse daily cigarettes			1.004	0.979	1.03	0.76		1.00	0.983	1.01	0.60

**Table 3:** Results from multivariate analysis of asthma phenotype (persistent and transient asthma).

	Persistent asthma							

BMI Trajectory groups	Females				Males			
	OR	95% CI		p value	OR	95% CI		p value
		Low	High			Low	High	

Increasing	4.09	1.04	16.15	**0.0442**	0.80	0.26	2.44	0.68
Decreasing	0.67	0.08	5.51	0.71	0.81	0.17	3.86	0.79
Stable	1				1			

	Transient asthma							

Increasing	1.56	0.82	2.95	0.17	0.75	0.45	1.23	0.25
Decreasing	1.46	0.43	4.95	0.52	0.67	0.29	1.53	0.33
Stable	1				1			

^*∗*^The following confounders were included in the multivariate analysis: age of child at baseline, father's age at birth, PMK current daily smoking status, PMK daily cigarettes, household ownership status, biological mother's history of asthma, and childhood allergy status.

## Data Availability

The National Longitudinal Survey of Children and Youths (NLSCY) used to support the findings of this study is available at Statistics Canada Research Data Center.
